# High-throughput, Label-Free Quantitative Proteomic Studies of the Anticancer Effects of Electrical Pulses with Turmeric Silver Nanoparticles: an *in vitro* Model Study

**DOI:** 10.1038/s41598-020-64128-8

**Published:** 2020-04-29

**Authors:** Lakshya Mittal, Ignacio G. Camarillo, Gowri Sree Varadarajan, Hemalatha Srinivasan, Uma K. Aryal, Raji Sundararajan

**Affiliations:** 1 School of Engineering Technology, Purdue University, West Lafayette, IN 47907 USA; 2 Department of Biological Sciences, Purdue University, West Lafayette, IN 47907 USA; 3 Purdue Center for Cancer Research, Purdue University, West Lafayette, IN 47907 USA; 4 Division of High Voltage Engineering, Dept. of Electrical & Electronics Engineering, College of Engineering, Anna University, Guindy, Chennai, TN 600025 India; 5 School of Life Sciences, B. S. Abdur Rahman Crescent Institute of Science & Technology, Chennai, TN 600048 India; 6 Purdue Proteomics Facility, Bindley Bioscience Center, Purdue University, West Lafayette, IN 47907 USA; 7 Department of Comparative Pathobiology, College of Veterinary Medicine, Purdue University, West Lafayette, IN 47907 USA

**Keywords:** Biotechnology, Cancer, Engineering

## Abstract

Triple negative breast cancer (TNBC) represents 15–20% of the over one million new breast cancer cases occurring each year. TNBC is an aggressive cancer phenotype, with low 5-year survival rates, high 3-year recurrence rates, and increased risk of metastasis. A lack of three commonly exploited hormone receptors renders TNBC resistant to endocrine therapies and lends to its critical absence of viable therapeutic targets. This necessitates the development of alternate and effective novel therapeutic strategies for TNBC. Towards this, our current work seeks to develop the technique of Electrical pulse (EP)-mediated Turmeric silver nanoparticles (TurNP) therapy, known as Electrochemotherapy (ECT), to effectively target TNBC cells. This technique involves the efficient delivery of natural bioactive molecules with anti-cancer effects via a biophysical means. In these experiments, the bioactive molecules are turmeric, a dried rhizome of *Curcuma longa* that has been used for centuries, both as a dietary supplement and as a medicine in Ayurveda (science of life) in the Indian subcontinent and in traditional Chinese medicine. Our results reveal the combined effect of TurNP + EP treatment in reducing MDA-MB-231 cell viability to as low as 9% at 12 h. Showing biological selectivity, this combination treatment has a substantially lower effect on non-tumorigenic mammary epithelial MCF10A cells (67% viability). To gain mechanistic insights into the actions of TurNP-based ECT treatment, we performed high-throughput, label-free quantitative proteomics studies. Proteomics results indicate that TurNP + EP treatment significantly influenced expression of a diverse list of proteins, including receptors, transcription factors, structural proteins, kinases, and metabolic enzymes. This include the downregulation of 25 proteins in PI3K-Akt signaling pathway (such as GRB2, EGFR, EPHA2, GNB1, GNB2, 14–3–3 family, and Integrin family proteins), and 12 proteins (AKR1A1, ALDOA, ALDOC, PGK1, PGM1, PGAM1, ENO1, ENO2, GAPDH, TPI1, LDHA, and LDHB) in the glycolytic pathway with concomitant reduction in metabolite levels (glucose uptake, and intracellular- lactate, glutamine, and glutamate). Compared to TurNP alone, TurNP + EP treatment upregulated 66 endoplasmic reticulum and 193 mitochondrial proteins, enhancing several processes and pathways, including Pyruvate Metabolism, Tricarboxylic acid (TCA) cycle, and Oxidative Phosphorylation (OXPHOS), which redirected the TNBC metabolism to mitochondria. This switch in the metabolism caused excessive production of H_2_O_2_ reactive oxygen species (ROS) to inflict cell death in MDA-MB-231 cells, demonstrating the potency of this treatment.

## Introduction

Triple Negative Breast Cancer (TNBC) is an aggressive and metastatic phenotype of breast cancer, which is clinically negative for estrogen (ER), progesterone (PR), and human epidermal growth factor receptor 2 (HER2) receptors^1^. In the absence of these receptors, TNBC is characterized by a critical lack of targeted therapies, as patients do not benefit from endocrine based therapies, resulting in poor survival and increased distance recurrences^2–4^. Hence, alternate therapies are sorely needed.

In efforts to address this need, novel electroporation techniques can represent a viable solution. The controlled local application of high intensity, short duration electrical pulses (EP) to cells leads to the structural rearrangement of phospholipids to generate temporary pores in the membrane by a phenomenon, deemed Electroporation^5,6^. Electroporation is widely used to enhance the uptake of external molecules, such as DNA, plasmids, drugs, and vaccines to bacteria, yeast, and mammalian cells. During electroporation, drug molecules can enter the cancer cells in various ways, such as diffusion, electro-osmotic, and colloid-osmotic flow^7^.

When electroporation is used together with chemotherapy drugs for their increased uptake, it is called Electrochemotherapy (ECT). ECT can dramatically increase the intracellular concentration and thus the cytotoxicity of several FDA approved drug molecules, such as bleomycin (1000-fold) and cisplatin (80-fold), leading to a complete tumor response at minimal drug dosages that by themselves show minimal/no anti-tumor activities and limit side-effects^8,9^.

ECT is widely used for advanced, inoperable, radiation- and chemo drug resistant patients in clinics across the European Union^10^. Campana *et al*. used ECT with bleomycin to treat tumors in 84 patients, who were unsuitable for standard therapies due to multiple, recurring or locally advanced basal cell carcinoma (BCC)^11^. In another multi-institutional study, Campana *et al*. successfully used ECT to treat 376 patients with carcinomas (breast, basal cell, and squamous cell), melanomas, sarcomas (Kaposi, and soft tissue), and other^12^. An overall response rate of 90.2% at 2 months and a complete response (CR) rate of 58.4% was obtained for 125 patients with breast cancer skin metastases in a multicenter study of ECT^13^. Recently, Kis *et al*. successfully performed ECT for challenging eyelid-periocular BCC conditions to obtain CR in advanced primary and recurrent cases of eyelid BCCs^14^. The advantage of ECT is that it has minimal effects on nearby healthy cells and tissues on the tumor margins, allowing an exceptional wound healing, and aesthetic and functional recovery post treatment^8,10,15,16^. Considering these excellent results, its safety, and affordability, ECT is now recommended for primary skin cancer and cutaneous metastases by several national and international guidelines^17,18^.

Typically, bleomycin, and occasionally, cisplatin, is used in ECT. In our current ECT study, we used TurNP as the chemodrug, as our research group interests include defining anticancer efficacy of natural compounds. Our previous studies have focused on combining Curcumin (diferuloylmethane)^19–22^, its nano-formulation, Nanocurcumin^23,24^, Turmeric^25^, Mentha Piperita (mint)^26^, and Holy Basil (Tulasi)^27^ with ECT against several types of cancer cells. We have shown that ECT enhanced the cytotoxicity of Curcumin treatment by 7-fold^19^, modulating multiple signaling pathways to increase apoptosis in MDA-MB-231 cells, while minimally effecting the viability of non-cancerous, MCF10A epithelial cells^20,21^. Curcumin constitutes 2–5% of dried Turmeric rhizome (*Curcuma longa*) and is the most extensively studied compound of Turmeric^28^. Turmeric has been used for centuries, both in cooking and as a medicine in Ayurveda (the science of life) in Indian subcontinent and also in traditional Chinese medicine. Turmeric is rich with various phytochemicals that have antioxidant, antibacterial, antiviral, anti-cancerous, anti-inflammatory properties and is useful to treat many diseases, including various cancers^28,29^. Although majority of the bioactivities of Turmeric are attributed to Curcumin, recent evidences suggest that other compounds of Turmeric can also show a potent bioactivity^28,29^. To increase the effectiveness of Turmeric, we synthesized and characterized the silver nanoparticles (TurNP) from an aqueous Turmeric extract. We demonstrated excellent antibacterial activities against ATCC 25922 and CTXM15 expressing antibiotic resistant *Escherichia coli*. TurNP also induced cell death in hormone receptor positive MCF7 and triple negative MDA-MB-231 breast cancer cells, while having minimal effects on the non-tumorigenic mammary epithelial MCF10A cells.

In the present work, we applied ECT with TurNP to further explore its efficacy against MDA-MB-231, human TNBC cells. First, we optimized the EP parameters for ECT and the TurNP dosage to maximize the MDA-MB-231 cell death, while sparing MCF10A cells. We showed that the combination of eight, 100µs duration EP of 800 V/cm with TurNP dosage of 15 µg/mL maximized the MDA-MB-231 cell death compared to TurNP and EP alone. Then we utilized label-free quantitative proteomics to better understand the anticancer effects. The hundreds of differentially expressed proteins elucidated the underlying molecular events of the enhanced effects of TurNP with ECT in MDA-MB-231 TNBC Cells. TurNP + EP suppressed several key proteins in multiple pathways, including PI3K-Akt signaling and glycolysis to direct the metabolism to mitochondria, which generated reactive oxygen species (ROS) that is associated with increased cell death.

## Results

### Cell viability and parameter optimization studies

**Figure 1:**
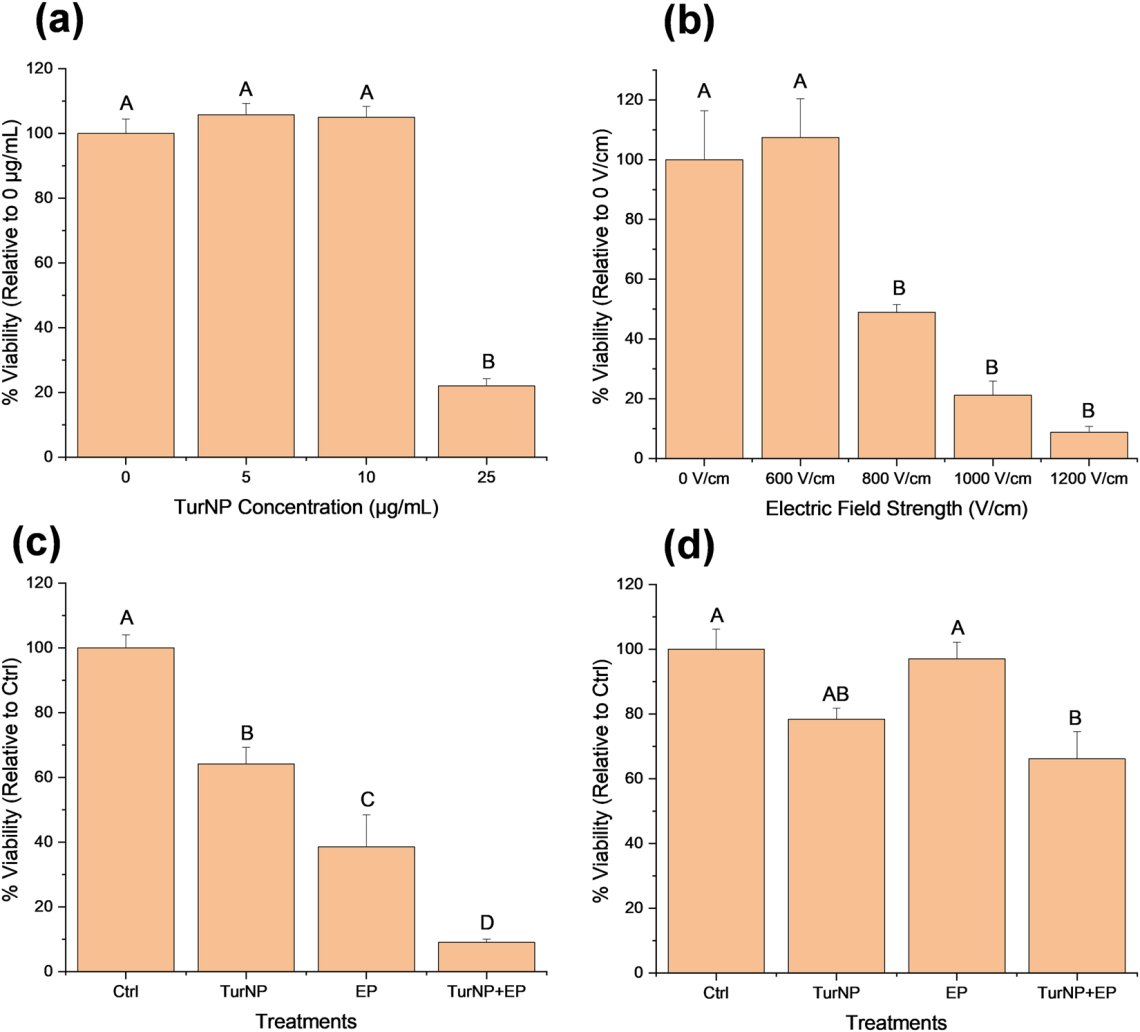
Cell viability studies at 12 h. (**a**) MDA-MB-231 cell viability at different concentrations of Turmeric Nanoparticles (TurNP) (N = 3). (**b**) MDA-MB-231 cell viability at different electric pulse (EP) parameters with varying electrical field strength (N=3). (**c,d**) Viability at the selected TurNP concentration (15 µg/mL) and EP parameter (800 V/cm), and their combination (TurNP + EP) for different cells (N = 4): (**c**) MDA-MB-231 cells, (**d**) MCF 10 A cells. Treated cells were incubated in the fresh media with RealTime-Glo MT Cell Viability reagent, and the luminescence was recorded at 12 h to quantify the viable cells relative to Control (Ctrl). The same letter or the same groups of letters indicate that they are not significantly different. The different letters or different group of letters indicate that they are significantly different (P < 0.05).

Figure 1a shows the cell viability of MDA-MB-231 cells for different dosages of TurNP (0, 5, 10, and 25 µg/mL) at 12 h, which were normalized with the viabilities of 0 µg/mL. (100%). The letter or group of letters tagged by Tukey’s test indicates their significance. For 5 µg/mL and 10 µg/mL, the viabilities were not significantly different from 0 µg/mL, indicating the limited effects of TurNP at lower dosages. However, the viability reduced significantly to 22% at the higher dosage of 25 µg/mL. Considering the higher cell death at 25 µg/mL, a moderate TurNP concentration of 15 µg/mL was selected for further studies.

Figure 1b shows the viability of MDA-MB-231 upon treatment with EP of various electric field strengths to obtain the optimal EP parameter. No cell death was observed at 600 V/cm, while increased cell deaths were observed at electric field strengths above 600 V/cm. The 12 h viability was 49% at 800 V/cm, and 21% at 1000 V/cm. It was just 9% at 1200 V/cm. From these, we selected 800 V/cm for the combinational studies.

Figure 1c shows the viability of MDA-MB-231 cells at 12 h for TurNP (15 µg/mL), EP (800 V/cm), and their combination (TurNP + EP). The results were normalized with the viability of Control (Ctrl) cells at 12 h (100%). The viability of TurNP treated cells decreased significantly to 64%, and to 39% for EP treatment. It dramatically reduced to 9% for TurNP + EP treatment, indicating the combined effect of TurNP + EP on effective cell death.

Figure 1d shows higher viability for the non-tumorigenic mammary epithelial MCF 10 A cells, for all treatments, demonstrating the minimal effect on nearby cells.

### Outline of Proteomics Studies and LC-MS/MS Reproducibility

**Figure 2:**
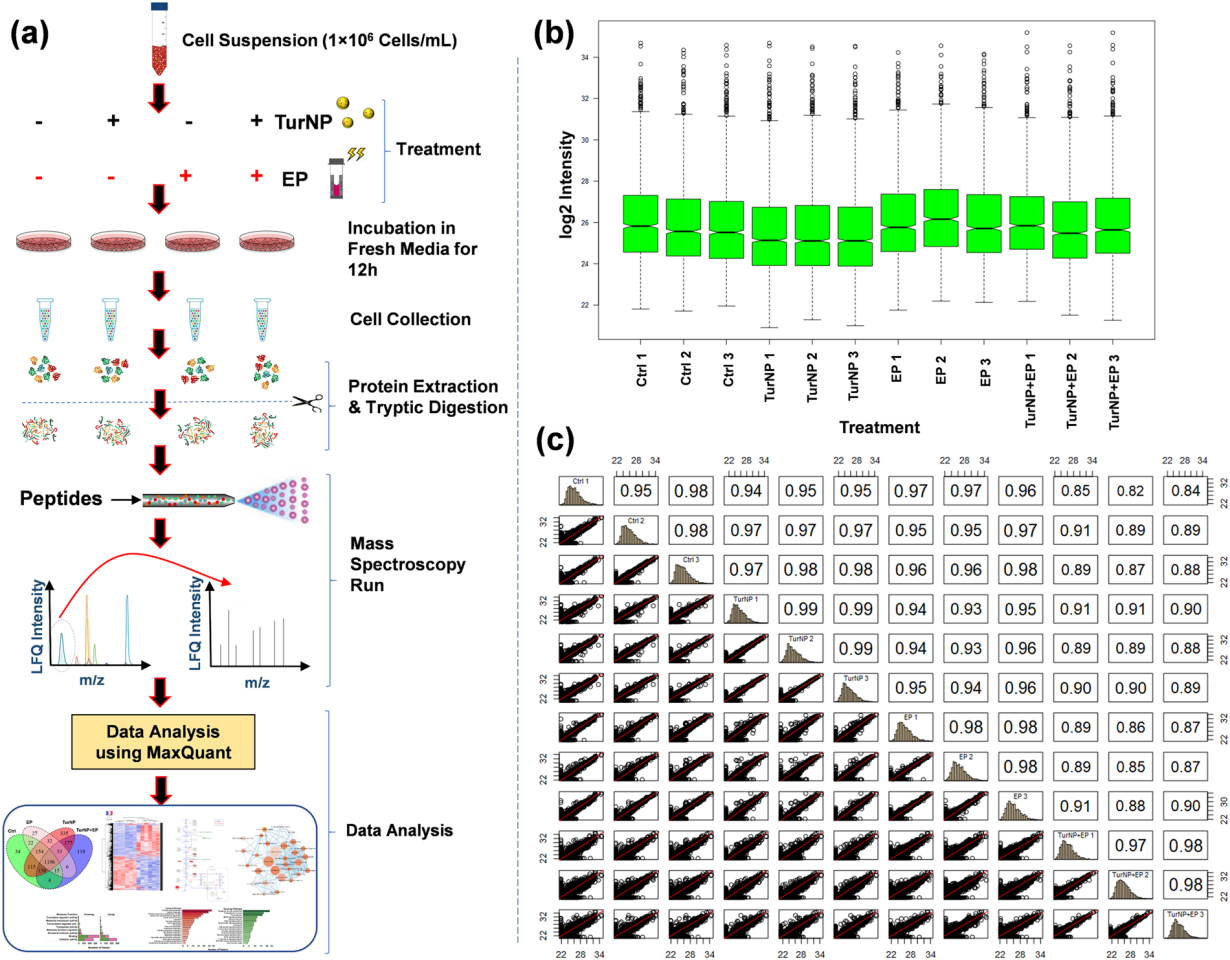
Experimental design of the proteomics studies. (**a**) The workflow: Ctrl and treated cells were incubated for 12 h in the fresh media, collected and lysed in urea and analyzed by LC-MS/MS for protein identification and quantification. (**b**) Boxplot of biological replicates of each treatment group. **(c**) Correlation plots of three biological replicates of all the treatments. R^2^ values are consistent and are noticeably higher between the replicates.

The experimental workflow of proteomics studies is shown in Fig. 2a, as explained in detail in Methods. In brief, the samples were run on the Q Exactive Orbitrap HF hybrid MS coupled with the UltiMate 3000 RSLCnano HPLC system to collect LC-MS/MS data. The tandem mass spectra were searched against the UniProt human protein database in MaxQuant. Proteins identified in at least two of the three biological replicates at 1% FDR and with at least 2 MS/MS (spectral) counts were considered for further analysis, resulting in unambiguous identification of 2426 proteins/protein families from 31823 peptides (Tables S1 and S2). The label-free quantification (LFQ) intensity was used to quantify the relative abundance of proteins for each sample.

Consistency of LFQ intensity is critical for the accurate measurement of protein abundances across multiple samples. Figure 2b shows the boxplot of the log2 LFQ intensity for the triplicate samples for each treatment. Here, the median and interquartile range were similar within the triplicates for a treatment, illustrating the consistency of the LC-MS/MS measurements across the replicates.

We also obtained high coefficient of determination (R^2^ ≥ 0.95) of LFQ intensities (Fig. 2c), indicating higher correlation between biological replicates from each treatment group than between replicates from different treatments.

**Figure 3:**
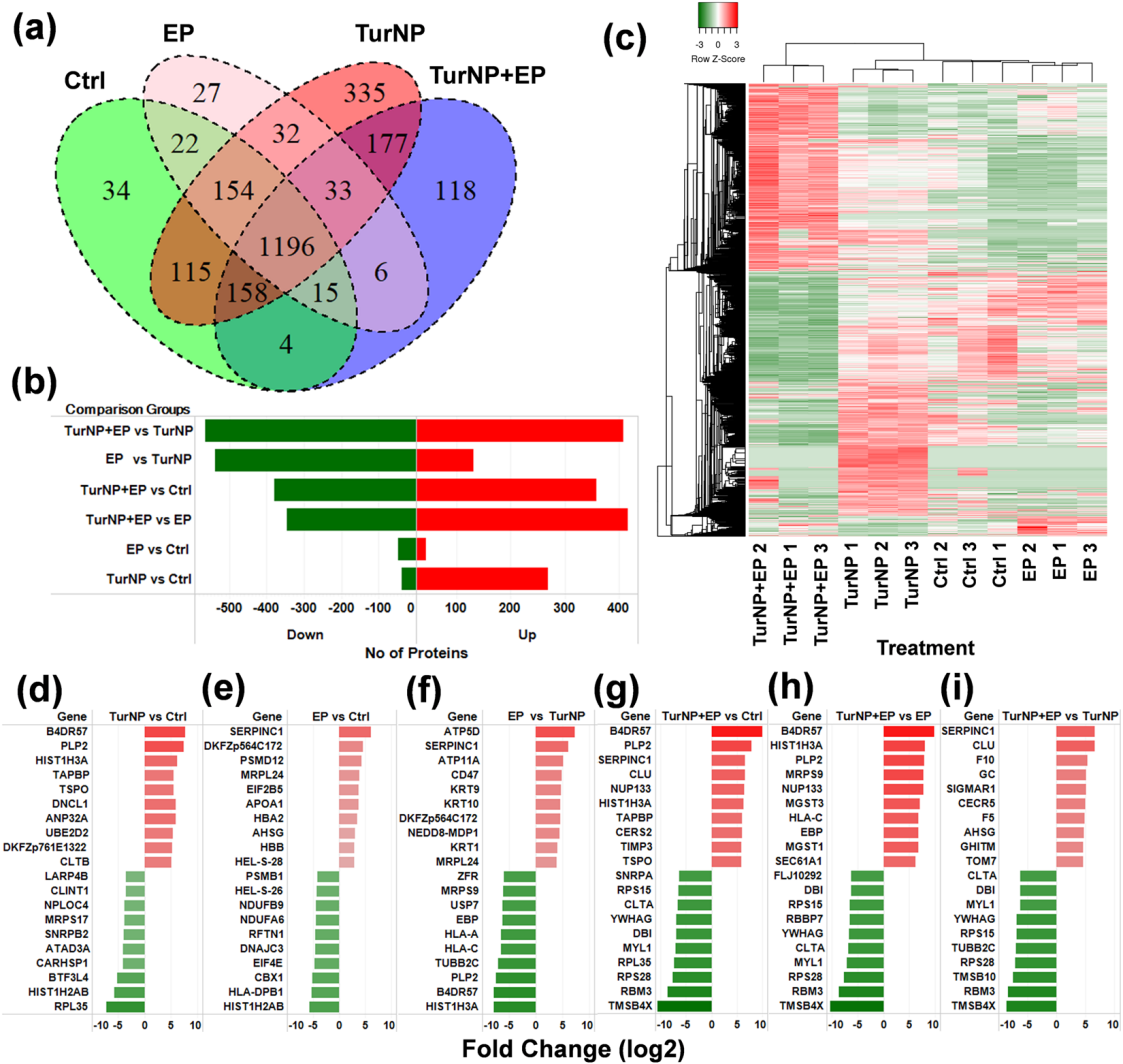
Overview of differentially regulated proteins. (**a**) Venn diagram showing the distribution of proteins in different treatment groups. (**b**) The number of significantly regulated (up and down) proteins in various pairwise comparisons. (**c**) Heatmap showing the expression levels of significantly regulated proteins in different treatments. LFQ intensities of proteins were clustered in rows and columns using Centroid Linkage method and Kendall’s Tau distance measurement method. Upregulated and downregulated proteins are represented with red and green colors, respectively. Heatmapper^72^ tool was used to generate this visualization. (**d–i**) The expression levels (log2 fold change) of top-10 significantly upregulated and downregulated proteins in different pairwise comparison groups: (**d**) TurNP vs Ctrl, (**e**) EP vs Ctrl, **(f**) EP vs TurNP, (**g**) TurNP + EP vs Ctrl. (**h**) TurNP + EP vs EP (**i**) TurNP + EP vs TurNP. Since B4DR57 is an uncharacterized protein and its gene name is not available, its Uniprot Accession number is used here.

Further, we used LFQ expression and MS/MS spectral count to classify the 2426 proteins into four treatment groups. A protein was considered to be present in a treatment, only if the LFQ was non-zero and MS/MS was 2 or higher (LFQ ≠ 0 and MS/MS ≥ 2) in at least two replicates among the triplicates. Figure 3a shows the Venn diagram classification of common and unique proteins present in each treatment. Among 2426 proteins, we identified 1698 proteins (69.9% of 2426) in Ctrl, 2200 (90.7% of 2426) in TurNP, 1485 (61.2% of 2426) in EP, and 1707 (70.4% of 2426) in TurNP + EP, of which 1196 proteins (49.3% of 2426) were common to all treatments (Table S2). The number of unique proteins were 34 in Ctrl, 335 in TurNP, 27 in EP, 118 in TurNP + EP. The highest number of total and unique proteins were found in TurNP. The reduction or loss in both the total and unique number of proteins in the case of TurNP + EP compared to TurNP suggests a substantial impact of TurNP + EP on cellular pathways.

### Overview of differentially regulated proteins

The LFQ values were used to calculate the differentially expressed proteins in six pairwise comparison groups: TurNP vs Ctrl, EP vs Ctrl, EP vs TurNP, TurNP + EP vs Ctrl, TurNP + EP vs EP, and TurNP + EP vs TurNP. For a comparison group, all the commonly and uniquely expressed proteins in both the samples were used to perform statistical analysis and to detect significant differentially expressed proteins (e.g. 2275 proteins were used in TurNP vs Ctrl).

Figure 3b shows the number of differentially expressed proteins in these comparison groups. Compared to the Ctrl, 269 proteins in the TurNP, 44 proteins in the EP, and 359 proteins in the TurNP + EP were upregulated (P < 0.05), and 39 proteins in the TurNP, and 49 in EP and 380 proteins in TurNP + EP were downregulated (P < 0.05) (Table S3). Compared to TurNP, 408 proteins were upregulated, and 564 were downregulated in the TurNP + EP treatment. Compared to EP, 416 proteins were upregulated, and 346 were downregulated in the TurNP + EP treatment, indicating that major changes observed for TurNP + EP treatment.

Heatmap was used to cluster and visualize the expression of these differentially regulated proteins (Fig. 3c). Protein expressions for triplicates within each treatment are clustered together and are consistent. Among treatments, clusters of proteins for Ctrl, TurNP, and EP are very close to each other, and are distinctly separate from the clusters of proteins for TurNP + EP, highlighting the difference in protein expression induced by TurNP + EP treatment.

Figure 3(d–i) show the top 10 significantly upregulated and downregulated proteins and their expression (log2 fold change) levels for 6 pairwise comparisons. The cDNA FLJ60818, a protein highly similar to complement C3 (Uniprot: B4DR57), of the immune system which plays a role in the development of inflammation^30^ was the most upregulated protein in TurNP vs Ctrl with a fold change of 7.77 (P = 2.8E^−07^), in TurNP + EP vs Ctrl and in TurNP + EP vs EP with a fold change of 9.95 (P = 1.3E^−07^) in each. The antithrombin-III (SERPINC1) was the most upregulated in EP vs Ctrl with a fold change of 6.18 (P = 6.5E^−06^), and in TurNP + EP vs TurNP with a fold change of 6.66 (P = 4.5E^−05^). The ATP synthase subunit delta, mitochondrial (ATP5D) was the most upregulated protein in TurNP vs EP with a fold change of 7.38 (P = 6.1E^−07^).

The 60S ribosomal protein L35 (RPL35) was the most downregulated protein in TurNP vs Ctrl with a fold change of 7.27 (P = 6.7E^−05^), while, histone H2A (HIST1H2AB) was the most downregulated protein in EP vs Ctrl with a fold change of 5.75 (P = 8.0E^−04^), and histone H3.1 (HIST1H3A) was the most downregulated protein with a fold change of 7.86 (P = 1.1E^−02^) in EP vs TurNP. The thymosin beta-4 (TMSB4X) was the most downregulated protein in TurNP + EP vs Ctrl with a fold change of 10.62 (P = 5.3E^−06^), in TurNP + EP vs EP with a fold change of 10.34 (P = 1.3E^−06^), and in TurNP + EP vs TurNP with a fold change of 8.61 (P = 1.9E^−07^).

### Functional annotation enrichment analysis of differentially regulated proteins

**Figure 4:**
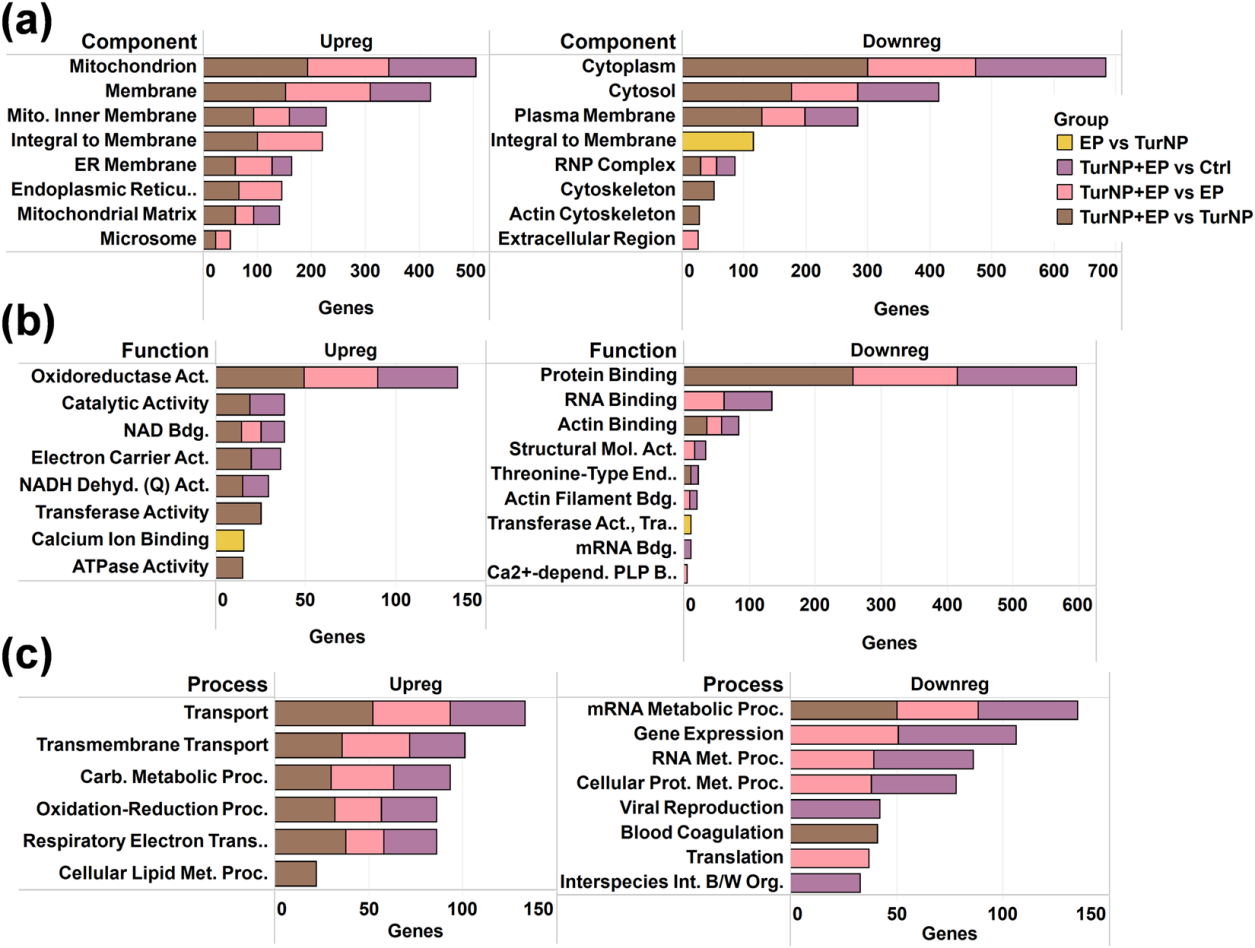
Key GO enrichment terms for differently regulated proteins in various comparisons. (**a**) Cellular component localization, (**b**) Molecular function, and (**c**) Biological process. A comprehensive list of the enriched GO terms obtained from Genecodis^70^ is presented in Table S4. No GO enrichment was found for the regulated proteins in TurNP vs Ctrl, and limited GO enrichment terms were found for the proteins regulated in EP vs Ctrl. Abbreviations - Act: Activity; Bdg: Binding, B/W: Between; Dehyd: Dehydrogenase; ER: Endoplasmic Reticulum; Int: Interaction; Met: metabolic; Org: Organisms; Proc: Process; Prot: Protein; Q: Ubiquinone; RNP: Ribonucleoprotein.

Further, we performed the Gene Ontology (GO) annotation analysis on differentially expressed proteins to identify their cellular component localizations, molecular functions, and biological processes (Fig. 4a–c) (Table S4). No GO enrichment was found for the regulated proteins in TurNP vs Ctrl, and 3 component, 2 function, and 5 process GO enrichment terms were found for the proteins regulated in EP vs Ctrl, indicating the low count of regulated proteins in these.

The upregulated proteins were primarily localized in mitochondrion, membrane, mitochondrial inner membrane, integral to membrane, endoplasmic reticulum (ER), ER membrane, mitochondrial matrix, and microsome for TurNP + EP compared to TurNP (Fig. 4a). Similar localization profile was also observed for proteins upregulated in TurNP + EP from Ctrl and EP. Majority of downregulated proteins in TurNP + EP vs TurNP were primarily localized in cytoplasm, cytosol, plasma membrane, and ribonucleoprotein (RNP) complex, as also observed for TurNP + EP vs Ctrl and TurNP + EP vs EP. However, only downregulated proteins for TurNP + EP vs TurNP were localized in cytoskeleton and actin cytoskeleton.

Molecular functional analysis showed higher representation of upregulated proteins related to oxidoreductase activity, catalytic activity, NAD binding, electron carrier activity, NADH dehydrogenase (Ubiquinone) activity, transferase activity, and ATPase activity for TurNP + EP (Fig. 4b). Only the calcium ion binding was upregulated for EP vs TurNP. The proteins involved in the protein binding, RNA binding, actin binding, structural molecule activity, actin filament binding, and mRNA binding were downregulated for TurNP + EP. The GO term transferase activity, transferring glycosyl groups, which is involved in catalysis of the transfer of a glycosyl group from one compound (donor) to another (acceptor) was downregulated for EP compared to TurNP.

The biological process analysis showed upregulation of proteins related to transport, transmembrane transport, carbon metabolic process, oxidation-reduction process, and respiratory electron transport chain in TurNP + EP compared to Ctrl, TurNP, and EP (Fig. 4c). This correlates well with the breakdown of cell membranes due to electrical pulses. The cellular lipid metabolic process was only upregulated for TurNP + EP compared to TurNP. The processes, such as, mRNA metabolic process, gene expression, RNA metabolic process, and cellular protein metabolic processes were downregulated for TurNP + EP compared to Ctrl and EP. The mRNA metabolic process and blood coagulation were downregulated for TurNP + EP compared to TurNP, while the translation was only downregulated for TurNP + EP from EP.

Collectively these results suggest that EP application with TurNP may upregulate the membrane proteins to facilitate the increased cellular transport process in MDA-MB-231 cells. Additionally, upregulation in organelle (mitochondrion and ER) proteins and activities, such as oxidoreductase, catalytic, NAD binding, NADH dehydrogenase (Ubiquinone), and electron carrier may activate intrinsic pathways to induce apoptotic cell death.

**Figure 5:**
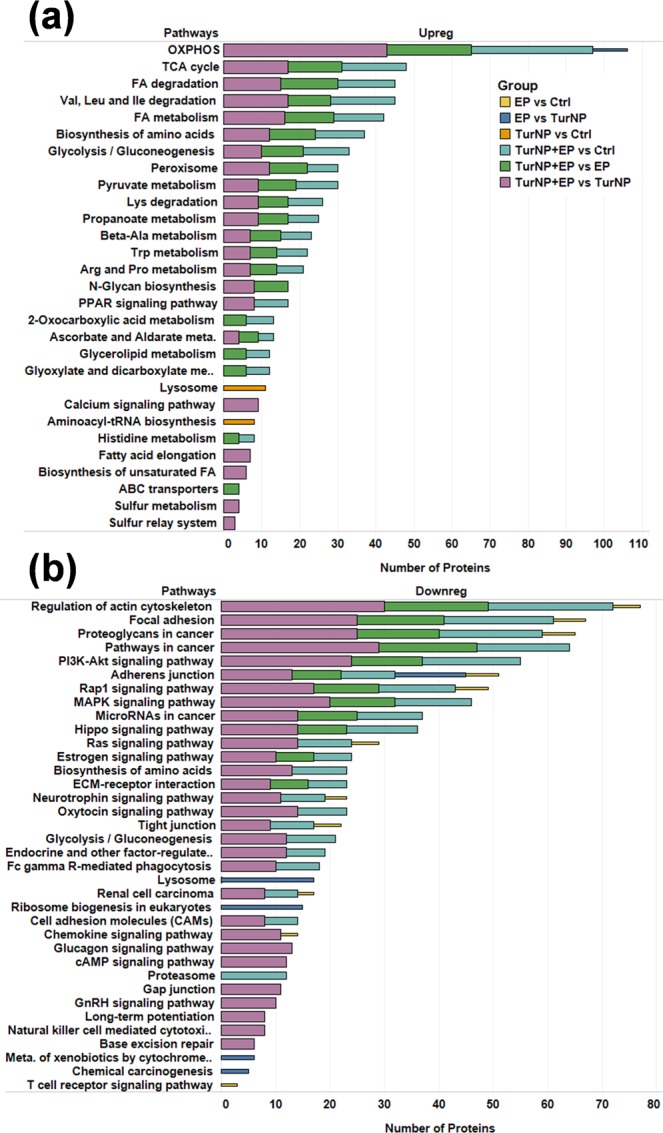
Key enriched pathways for differently regulated proteins in various comparisons using DAVID 6.8^67,68^. (**a**) Upregulated, and (**b**) Downregulated. The comprehensive list of enriched pathways is presented in Table S5. Abbreviations - Ala: Alanine; Asp: Aspartate; Deg: Degradation; FA: Fatty Acid; Ile: Isoleucine; Leu: Leucine, Lys: Lysine; Trp: Tryptophan; Val: Valine.

Additionally, KEGG pathway enrichment for upregulated proteins showed (Fig. 5a) enrichment of oxidative phosphorylation (OXPHOS), tricarboxylic acid (TCA) cycle, fatty acid (FA) degradation, FA metabolism, amino acid (valine, leucine, isoleucine, and lysin) degradation, glycolysis, peroxisome, pyruvate metabolism, and aromatic amino acid (tryptophan) metabolism in TurNP + EP compared to Ctrl, TurNP and EP. The OXPHOS was also upregulated for EP from TurNP. The calcium signaling pathway, fatty acid elongation, biosynthesis of unsaturated FA, sulfur metabolism, and sulfur relay system were uniquely upregulated for TurNP + EP from TurNP. Also, ATP-binding cassette (ABC) transporters pathway which activates the transport of various substrates (ions, drugs, protein, peptides, lipids, sugars, and sterols) was uniquely upregulated in TurNP + EP from EP, which included the proteins ATP binding cassette subfamily B member 7(ABCB7, an iron transporter^31^), ATP binding cassette subfamily D member 3 (ABCD3), transporter 1, ATP binding cassette subfamily B member(TAP1), transporter 2, ATP binding cassette subfamily B member(TAP2). The lysosome, and aminoacyl-tRNA biosynthesis pathways were upregulated only in TurNP from Ctrl.

The pathways responsible for increased cell proliferation, differentiation, migration, survival, and evasion of cell death and apoptosis were downregulated for TurNP + EP (Fig. 5b). Among these pathways, the regulation of actin cytoskeleton, focal adhesion, PI3K-Akt signaling, adherens junction, Rap1 signaling, MAPK signaling, MicroRNAs in cancer, and hippo signaling pathways were downregulated for TurNP + EP compared to Ctrl, TurNP, and EP. Additionally, RAS signaling, neurotrophin signaling, tight junction, and glycolysis were downregulated in TurNP + EP only from Ctrl, and TurNP. The regulation of actin cytoskeleton, focal adhesion, adherens junction, Rap1 signaling, RAS signaling, neurotrophin signaling, and tight junction pathways were also downregulated for EP from Ctrl, however with less proteins compared to TurNP + EP, highlighting the combined effects of TurNP with EP. The T cell receptor signaling pathway was uniquely downregulated in EP compared to Ctrl.

### Impact on PI3K-Akt signaling and glycolysis pathways

**Figure 6:**
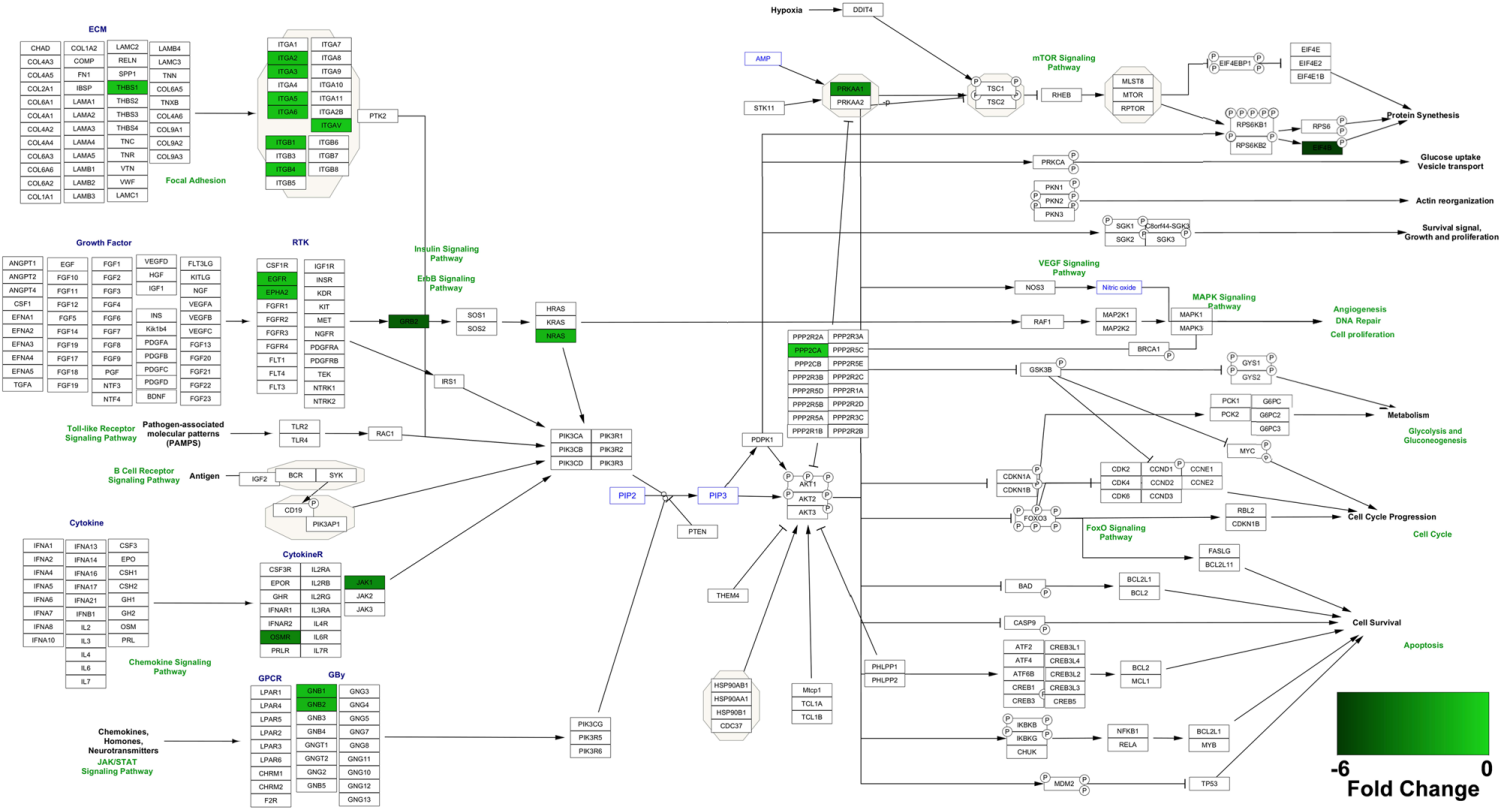
Identification of key PI3K-Akt Signaling Pathway downregulated proteins in TurNP + EP compared to TurNP in MDA-MB-231 cells. Proteins from GO term “PI3K-Akt Signaling Pathway” from KEGG^66^ pathway analysis were uploaded to the Cytoscape 3.6.1 software^69^ and matched to the PI3K-Akt Signaling Pathway using the WikiPathway app (beta), with the degree of shading representing the fold change.

Since PI3K is one of the most commonly mutated pathways in TNBC^32^, which could be targeted therapeutically, as suggested by phase II trials^33^, we next examined the downregulated proteins in PI3K-Akt signaling pathway to understand the extent of its inhibition upon TurNP + EP treatment. Table 1 shows the list of 25 significantly downregulated PI3K-Akt pathway proteins in at least one comparison, and their fold change (log2). Figure 6 shows the visualization of PI3K-Akt pathway populated with the downregulated proteins in TurNP + EP vs TurNP comparison and their relative expression levels. Among these proteins, the growth factor receptor-bound protein 2 (GRB2) was the most downregulated protein (TurNP + EP vs Ctrl - ↓4.82×, P = 2.6E^−07^; TurNP + EP vs TurNP - ↓4.72×, P = 2.3E^−07^). The GRB2 can directly interact with receptor tyrosine kinases (RTKs), such as epidermal growth factor receptor (EGFR) to activate the downstream of several oncogenic signaling pathways, including PI3K-Akt and RAS signaling (Fig. 6)^34^. The EGFR, the oncogenic cell-surface RTK^35^ ephrin type-A receptor 2 (EPHA2), as well as the Guanine nucleotide-binding protein G(I)/G(S)/G(T) subunit beta-1 (GNB1) and beta-2 (GNB2), G family proteins were downregulated in TurNP + EP compared to other treatments.

**Table 1:** The expression levels of all downregulated proteins in PI3K-Akt signaling pathway for at least one of the three comparisons.

S.N.	Uniprot Access. No.	Protein	Gene Name	TurNP + EP vs Ctrl		TurNP + EP vs EP		TurNP + EP vs TurNP	
				Fold Change	P Value	Fold Change	P Value	Fold Change	P Value
1	P29317	Ephrin type-A receptor 2	EPHA2	−0.66^#^	6.9E-03*	−0.48	2.5E-02*	−1.03^#^	1.7E-03*
2	P62879	Guanine nucleotide-binding protein G(I)/G(S)/G(T) subunit beta-2	GNB2	−1.16^#^	4.5E-04*	−0.83^#^	3.3E-03*	−1.35^#^	7.6E-04*
3	P00533	Epidermal growth factor receptor	EGFR	−0.67^#^	8.4E-03*	−0.64^#^	9.0E-03*	−0.95^#^	1.4E-03*
4	P23588	Eukaryotic translation initiation factor 4B	EIF4B	−5.43^#^	6.0E-06*	−5.14^#^	4.6E-08*	−5.83^#^	2.4E-08*
5	P62993	Growth factor receptor-bound protein 2	GRB2	−4.82^#^	2.6E-07*	−3.12^#^	1.2E-01	−4.72^#^	2.3E-07*
6	P17301	Integrin α2	ITGA2	−0.62^#^	1.3E-02*	−0.42	2.9E-03*	−0.97^#^	6.9E-04*
7	P26006	Integrin α3	ITGA3	−0.80^#^	2.5E-03*	−0.68^#^	1.1E-03*	−0.78^#^	1.6E-03*
8	P23229	Integrin α6	ITGA6	−0.71^#^	1.7E-03*	−0.91^#^	3.6E-04*	−0.82^#^	8.9E-04*
9	P05556	Integrin β1	ITGB1	−0.85^#^	1.8E-03*	−0.67^#^	1.1E-04*	−0.91^#^	2.1E-04*
10	P16144	Integrin β4	ITGB4	−0.53^#^	5.7E-05*	−0.46	6.7E-03*	−0.66^#^	4.8E-04*
11	P01111	GTPase NRas	NRAS	−1.11^#^	1.3E-03*	−0.78^#^	6.2E-03*	−1.28^#^	1.7E-03*
12	P67775	Serine/threonine-protein phosphatase 2A catalytic subunit alpha isoform	PPP2CA	−0.54^#^	4.1E-03*	−0.49	2.6E-03*	−0.53^#^	4.7E-03*
13	P07996	Thrombospondin-1	THBS1	−1.19^#^	2.0E-04*	−0.68^#^	6.3E-04*	−0.79^#^	3.5E-04*
14	P31946	14-3-3 protein beta/alpha	YWHAB	−1.78^#^	1.2E-02*	−1.51^#^	1.5E-02*	−1.71^#^	8.5E-03*
15	P62258	14-3-3 protein epsilon	YWHAE	−1.54^#^	4.3E-03*	−1.37^#^	3.2E-03*	−1.57^#^	7.4E-04*
16	P61981	14-3-3 protein gamma	YWHAG	−6.84^#^	9.1E-06*	−6.66^#^	1.4E-05*	−6.88^#^	1.6E-07*
17	P27348	14-3-3 protein theta	YWHAQ	−0.84^#^	4.3E-02*	−0.52^#^	6.8E-02	−1.07^#^	1.9E-03*
18	P63104	14-3-3 protein zeta/delta	YWHAZ	−1.77^#^	9.2E-03*	−1.55^#^	6.1E-03*	−1.81^#^	3.0E-03*
19	P11047	Laminin subunit gamma-1	LAMC1	−0.42^#^	4.7E-03*	−0.77^#^	3.7E-04*	−0.24	7.7E-03*
20	P62873	Guanine nucleotide-binding protein G(I)/G(S)/G(T) subunit beta-1	GNB1	0.01	9.5E-01	0.28	9.1E-02	−1.07^#^	3.2E-03*
21	P23458	Tyrosine-protein kinase JAK1	JAK1	0.00	N/A	0.00	N/A	−3.02^#^	3.7E-07*
22	P08648	Integrin α5	ITGA5	−0.28	2.0E-02*	−0.34	5.7E-02	−0.53^#^	2.2E-02*
23	P06756	Integrin αV	ITGAV	−0.04	6.0E-01	1.23^#^	4.0E-01	−0.64^#^	1.4E-03*
24	Q99650	Oncostatin-M-specific receptor subunit beta	OSMR	0.00	N/A	0.00	N/A	−3.17^#^	1.3E-05*
25	P54646	5-AMP-activated protein kinase catalytic subunit alpha-2	PRKAA2	0.00	N/A	0.00	N/A	−2.62^#^	1.1E-05*

The proteins with fold change indicated with ^#^(|fold change | ≥0.5) and P value with *(P < 0.05) only are significantly regulated for a comparison.

**Figure 7:**
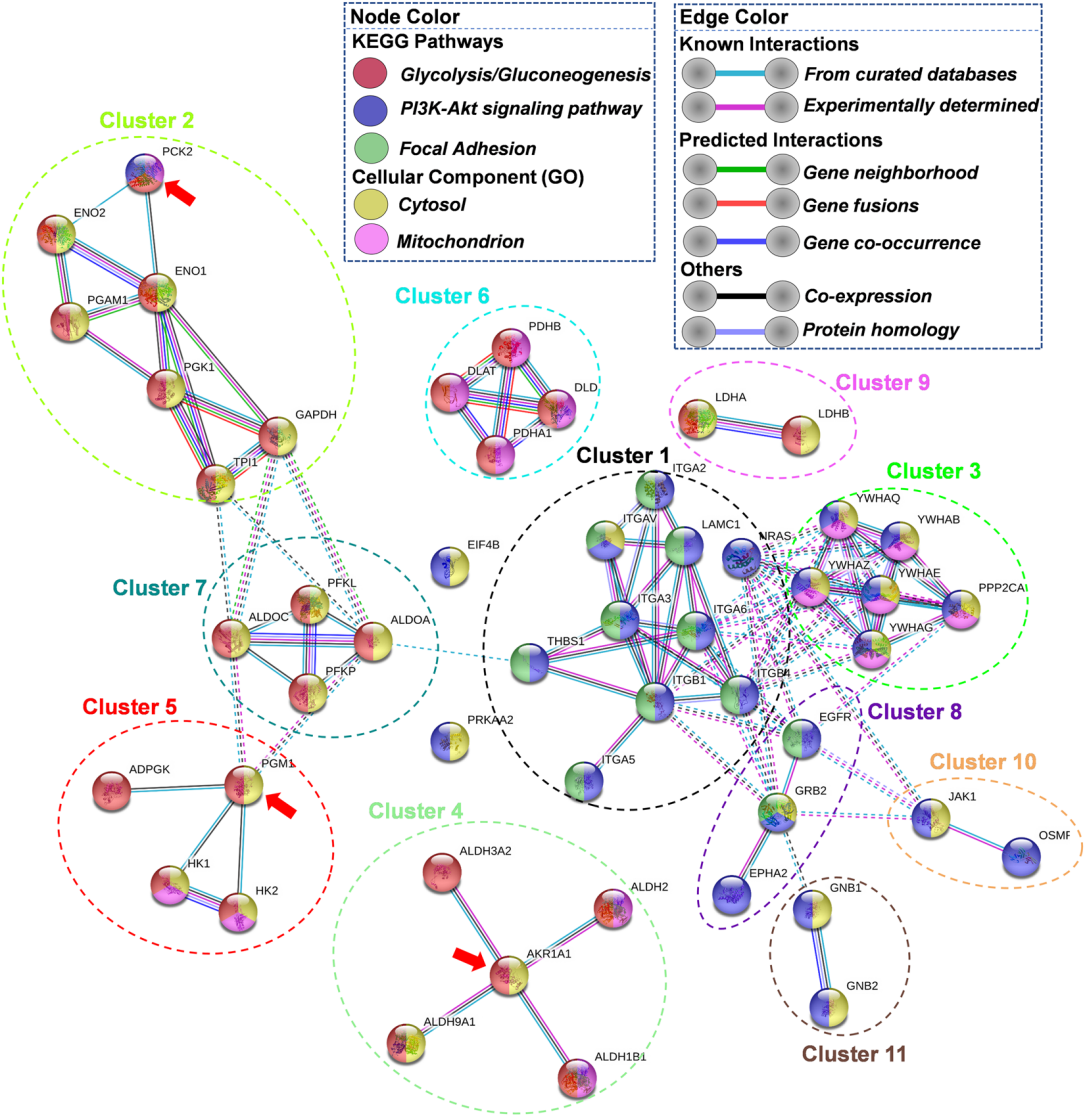
The String interaction analysis of the significantly regulated proteins in PI3K-Akt signaling and glycolysis pathways. The significantly regulated proteins from these pathways were uploaded to STRING^71^ tool to visualize the interaction and functional enrichment with evidence as meaning of network edges, and active interaction sources to be Experiments, Database, Co-expression, Neighborhood, Gene Fusion, and Co-occurrence, with minimum required interaction score as highest confidence (0.9). An MCL clustering analysis with 3 as inflation parameter was run on the network nodes to cluster them in different groups. Here the node color represents the KEGG^66^ pathways and the localization of these proteins. The color of the edges is based on the interactions: (known, predicted, or other). Red arrows indicate the occasional outliers in a cluster from the other (up/downregulated) group for regulated proteins in glycolysis.

Additionally, the 14–3-3 family proteins, including 14–3-3 beta/alpha (YWHAB), 14–3-3 epsilon (YWHAE), 14-3-3 protein gamma (YWHAG), 14–3-3 theta (YWHAQ), and 14–3-3 zeta (YWHAZ), as well as integrin family proteins (integrins α2, α3, α5, α6, αV, β1, and β4) were also downregulated for TurNP + EP (Table 1). The most common beta subunit in integrin heterodimers is β1 can bind to many α subunits (as shown in Fig. 7) to play key roles in migration and stemness^36,37^. This was studied using STRING analysis.

STRING interaction and clustering analysis (Fig. 7) of downregulated proteins including PI3K-Akt pathway proteins (blue color nodes) and glycolytic enzymes (up and down both - red color nodes) indicate that all the integrins family proteins are clustered together and β1 interacts directly with α2, α3, α5, α6, αV, and β4 (cluster #1). All the integrins together with EGFR, and GRB2 also participate in focal adhesion pathway, as indicated by green color nodes, and other downregulated PI3K-Akt pathway proteins, Laminin subunit gamma-1 (LAMC1) and Thrombospondin-1 (THBS1) were also clustered together with integrins. THBS1 is also linked with glycolysis, as highlighted by its interaction with aldolase A (ALDOA).

Figure 7 also demonstrates that 26 differentially regulated glycolysis pathway proteins form 6 different clusters (clusters # 2, 4, 5, 6, 7, and 9). Among these proteins, 14 were upregulated, and 12 were downregulated in TurNP + EP for at least one of the three pairwise comparison groups. Table 2 lists these proteins and their expressions.

**Table 2:** The expression levels of all significantly up- and downregulated proteins in glycolysis pathway for at least one of the three comparisons.

S.N.	Uniprot Access. No.	Protein	Gene Name	TurNP + EP vs Ctrl		TurNP + EP vs EP		TurNP + EP vs TurNP	
				Fold Change	P Value	Fold Change	P Value	Fold Change	P Value
1	Q9BRR6	ADP-dependent glucokinase	ADPGK	4.09^#^	1.1E-06*	4.09^#^	1.1E-06*	0.28	5.1E-02
2	P30837	Aldehyde dehydrogenase X, mitochondrial	ALDH1B1	0.82^#^	1.0E-03*	0.60^#^	1.4E-02*	0.77^#^	7.3E-04*
3	P05091	Aldehyde dehydrogenase, mitochondrial	ALDH2	0.89^#^	3.1E-04*	0.71^#^	3.7E-03*	0.83^#^	3.7E-03*
4	P51648	Aldehyde dehydrogenase family 3 member A2	ALDH3A2	4.93^#^	2.4E-07*	4.93^#^	2.4E-07*	0.97^#^	9.7E-04*
5	P49189	4-trimethylaminobutyraldehyde dehydrogenase	ALDH9A1	5.28^#^	3.5E-07*	5.28^#^	3.5E-07*	0.36	1.3E-02*
6	Q86YI5	Acetyltransferase component of pyruvate dehydrogenase complex	DLAT	0.87^#^	2.0E-04*	0.71^#^	3.2E-03*	1.10^#^	4.9E-05*
7	P19367	Hexokinase-1	HK1	0.65^#^	8.7E-02	0.76^#^	3.3E-03*	0.36	2.4E-02*
8	P52789	Hexokinase-2	HK2	4.87^#^	6.8E-03*	5.77^#^	3.0E-03*	3.07^#^	5.7E-03*
9	Q01813	ATP-dependent 6-phosphofructokinase, platelet type	PFKP	1.17^#^	5.3E-03*	1.11^#^	3.5E-03*	0.75^#^	4.4E-03*
10	P08559	Pyruvate dehydrogenase E1 component subunit alpha, somatic form, mitochondrial	PDHA1	1.16^#^	1.9E-04*	1.26^#^	6.6E-04*	0.95^#^	6.3E-03*
11	P11177	Pyruvate dehydrogenase E1 component subunit beta, mitochondrial	PDHB	0.70^#^	2.5E-03*	0.54^#^	1.5E-02*	0.76^#^	1.6E-03*
12	P09622	Dihydrolipoyl dehydrogenase, mitochondrial	DLD	0.16	2.1E-01	0.03	7.5E-01	0.69^#^	4.7E-03*
13	P17858	ATP-dependent 6-phosphofructokinase, liver type	PFKL	0.86^#^	1.8E-02*	0.61^#^	6.1E-02	3.69^#^	2.4E-02*
14	Q16822	Phosphoenolpyruvate carboxykinase [GTP], mitochondrial	PCK2	0.67^#^	1.5E-03*	0.49	5.2E-02	2.21^#^	2.0E-01
15	P14550	Aldo-keto reductase family 1 member A1	AKR1A1	−4.16^#^	7.3E-05*	0.00	N/A	-4.52^#^	2.2E-07*
16	P04075	Fructose-bisphosphate aldolase A	ALDOA	−1.87^#^	5.3E-03*	−1.70^#^	5.4E-04*	−1.68^#^	4.9E-04*
17	P09972	Fructose-bisphosphate aldolase C	ALDOC	−1.44^#^	3.7E-01	0.00	N/A	−4.44^#^	6.9E-07*
18	P06733	Alpha-enolase	ENO1	−1.68^#^	4.8E-03*	−1.08^#^	1.4E-03*	−1.67^#^	7.4E-05*
19	P09104	Gamma-enolase	ENO2	−5.18^#^	3.7E-05*	−3.65^#^	1.2E-01	−4.63^#^	4.3E-06*
20	P04406	Glyceraldehyde-3-phosphate dehydrogenase	GAPDH	−0.73^#^	5.7E-02	−0.34	2.2E-02*	−0.51^#^	1.0E-03*
21	P00338	Lactate dehydrogenase A chain	LDHA	−0.79^#^	8.3E-03*	−0.15	2.8E-01	−1.07^#^	4.6E-04*
22	P07195	Lactate dehydrogenase B chain	LDHB	−1.55^#^	1.8E-03*	−1.19^#^	2.0E-03*	−1.49^#^	2.7E-04*
23	P36871	Phosphoglucomutase-1	PGM1	−2.10^#^	7.9E-03*	−1.52^#^	9.1E-03*	−2.06^#^	3.5E-03*
24	P00558	Phosphoglycerate kinase 1	PGK1	−0.91^#^	5.7E-02	−0.35	3.5E-02*	−0.96^#^	1.2E-03*
25	P18669	Phosphoglycerate mutase 1	PGAM1	−1.91^#^	1.1E-02*	−1.57^#^	1.1E-02*	−1.84^#^	6.0E-03*
26	P60174	Triosephosphate isomerase	TPI1	−2.17^#^	6.5E-03*	−2.05^#^	4.3E-03*	−1.87^#^	3.3E-03*

The proteins with fold change indicated with ^#^(|fold change | ≥0.5) and P value with *(P < 0.05) only are significantly regulated for a comparison.

The clustering analysis also highlights that the majority of upregulated and downregulated proteins are clustered together in separate clusters, with red arrows indicating the occasional outliers in a cluster from the other (up/downregulated) group. Clusters 4, 5, and 6 represent upregulated proteins, and clusters 2, and 9 represent downregulated proteins, while cluster 7 represents the mixed population, with 2 proteins from each group (PFKL and PFKP: upregulated, ALDOA and ALDOC: downregulated).

The localization studies highlight that while downregulated proteins are cytosolic (yellow color nodes), most of the upregulated proteins are mitochondrial (pink color nodes) correlating well with the global enrichment trends for the regulated proteins as shown previously in Figs. 4 and 5.

**Figure 8:**
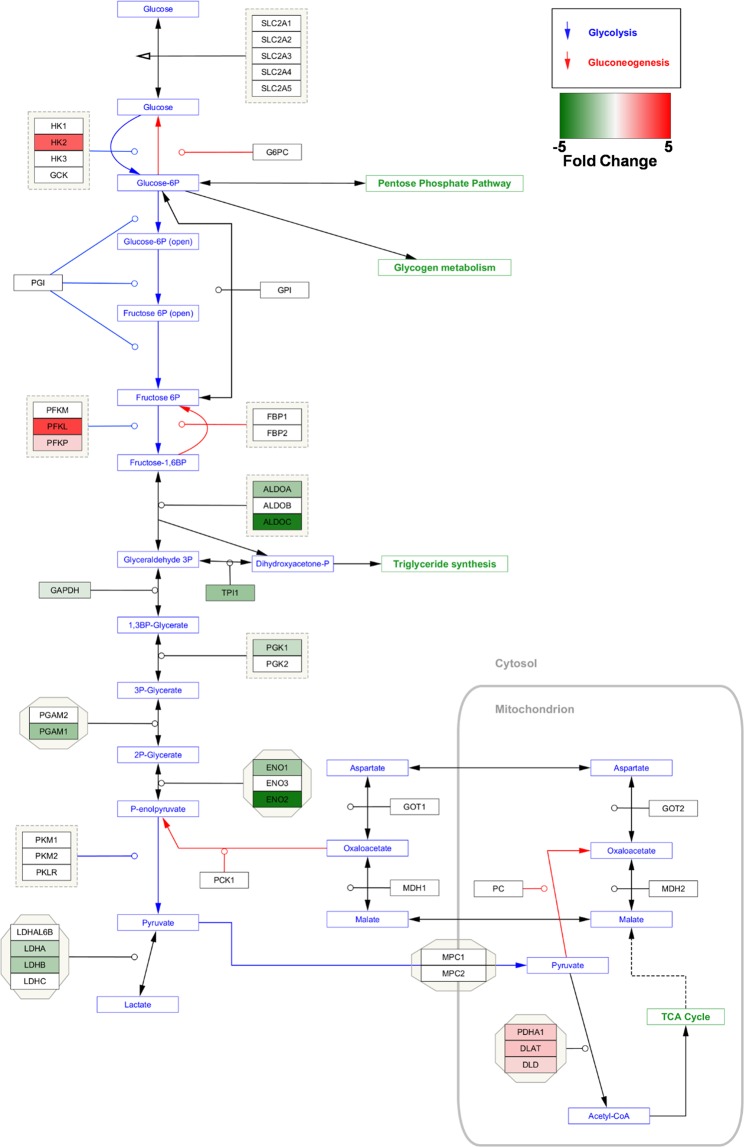
Identification of key glycolysis pathway proteins downregulated (green) and upregulated (red) in TurNP + EP compared to TurNP in MDA-MB-231 cells. Proteins from GO term “glycolysis/gluconeogenesis” from KEGG^66^ pathway analysis were uploaded to the Cytoscape 3.6.1 software^69^ and matched to the glycolysis pathway using the WikiPathway app (beta), with the degree of shading representing the fold change.

The identification and expression of key significantly regulated glycolytic proteins for TurNP + EP compared to TurNP is shown in Fig. 8.

### Validation of proteomics results

**Figure 9:**
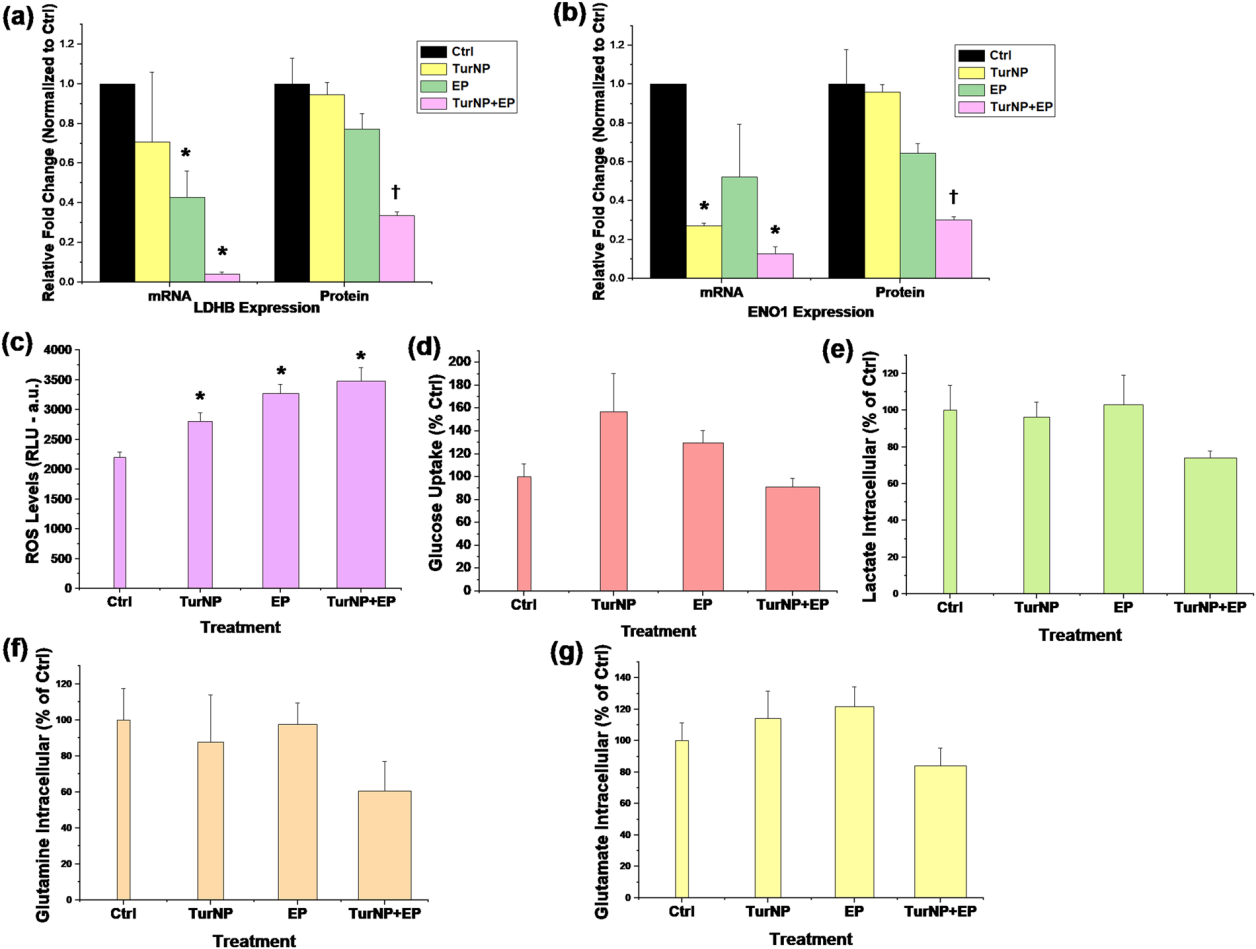
The validation experiments. (**a,b**) The mRNA and protein level expressions of LDHB and ENO1 in MDA-MB-231 cells for different treatment conditions at 12 h (N = 3): (**a**) LDHB, (**b)** ENO1. The ∆∆Cq method was used to determine relative mRNA expression levels at 12 h of the treatment from qPCR data with 18 S ribosomal RNA (18 s rRNA) as an internal reference. The protein expression levels were recorded at 12 h of the treatment using proteomics (N = 3). *P  <  0.05-mRNA levels significantly different from Ctrl. ^†^P  <  0.05-protein levels significantly different from Ctrl. (**c**) The H_2_O_2_ ROS levels in MDA-MB-231 cells at 12 h of treatment (N = 4). (d-g) Levels of various metabolites in MDA-MB-231 cells for different treatments at 12 h (N = 3) (**d**) Glucose uptake, (**e**) Intracellular Lactate, (**f**) Intracellular glutamine, (**g**) Intracellular glutamate. Error bars are calculated using standard error. *P  <  0.05-metabolites levels significantly different from Ctrl.

The proteomics results were validated by verifying the correlation between protein level changes and the transcription level changes for the glycolytic genes LDHB and ENOI. Their mRNA levels were checked using using real time quantitative PCR (qPCR) after 12 h of treatment. Figure 9a,b shows these results. The mRNA/protein levels were normalized with Ctrl mRNA/protein expression levels (level 1). The mRNA expression of LDHB was 0.71 TurNP, while it reduced to 0.43 for EP and 0.04 in TurNP + EP (Fig. 9a). In comparison, the protein expression of LDHB was 0.95 for TurNP, while it reduced to 0.77 for EP and to 0.34 for TurNP + EP treatment.

For ENO1, the mRNA expression was the lowest at 0.13 for TurNP + EP, which was also the case with the protein level at 0.3 (Fig. 9b). These results demonstrate good correlation between mRNA and protein levels for LDHB and ENO1 and validate that these genes are downregulated at transcription levels upon TurNP + EP treatment.

Figure 9c shows the average luminescence (Lum) as relative light units (RLU), which represents the H_2_O_2_ reactive oxygen species levels in MDA-MB-231 cells at 12 h. The Lum was 2199 for Ctrl, which increased significantly to 2800 for TurNP treatment and to 3266 for EP treatment. The highest Lum of 3481 was observed for TurNP + EP, a 1.6-fold and significant increase in H_2_O_2_ levels compared with Ctrl. We also quantified the uptake and intracellular levels of key metabolites, such as glucose uptake, intracellular lactate, glutamine, and glutamate (Fig. 9d–g) that suggest a decreasing trend in the metabolites levels upon TurNP + EP treatment, compared to the Ctrl.

## Discussion

In this study, we sought to better understand the anticancer effects of TurNP + EP on human, MDA-MB-231 TNBC cells, utilizing cell viability and quantitative proteomic studies. The viability studies showed that this combined treatment substantially promoted tumor cell death (91%) at 800 V/cm and 15 µg/mL TurNP.

Proteomic results revealed that TurNP + EP influenced the expressions of many proteins belonging to multiple cellular components, molecular functions, and biological processes. Among the differentially regulated proteins, we identified receptors, transcription factors, structural proteins, kinases, and metabolic enzymes. The majority of upregulated proteins in TurNP + EP treatment belonged to organelles (ER, and mitochondrion) and membrane structures, while the majority of downregulated proteins resided in cytosol, cytoskeleton, and extracellular regions.

TurNP + EP treatment downregulated 25 key PI3K-Akt pathway proteins (Table 2). This effect may have particular significance given that PI3K is one of the most commonly mutated pathways in TNBC^32,33^. The GRB2 is overexpressed in breast cancers, and is a key molecule in intracellular signal transduction, which can directly interact with RTKs, such as epidermal growth factor receptor (EGFR) to activate several downstream oncogenic signaling pathways, including PI3K-Akt and RAS signaling^34^. The EGFR is frequently overexpressed and it enhances aerobic glycolysis and is linked with poor prognosis in TNBC^38–40^. The EPHA2 is uniquely upregulated in TNBC, and its silencing impairs cell growth and stimulates apoptosis^35^. Further, the G family proteins, GNB1 and GNB2 are implicated in cancer proliferation, survival, invasion, metastasis, survival and resistance^41,42^. The 14-3-3 family proteins play important role in regulating multiple signaling pathways, including cell cycle, autophagy, apoptosis, and glycolysis^43^. These proteins are also downregulated in response to EP with curcumin in MDA-MB-231 cells^20^.

Other major changes in protein expression due to TurNP + EP treatment were also observed for proteins involved in glycolysis, TCA cycle and OXPHOS. Majority of key glycolytic enzymes were downregulated in agreement with previous reports^44–53^. For example, ENO1 is a highly expressed TNBC biomarker and its function is positively related with the distinct TNBC metabolism^44,45^. While LDHA is upregulated in variety of cancers, LDHB is specifically upregulated in basal-like TNBC and is an essential gene in TNBC. Patients with elevated LDHB levels in breast tumors show a poor clinical outcome^46^. LDHB may play a critical role in reverse Warburg effect^47^, an alternate way by which hypoxic and glucose-deprived cells actively utilize lactate secreted from neighboring cells undergoing aerobic glycolysis^46–50^. Downregulation of ALDOA, PGK1, and several other glycolytic enzymes listed in Table 2 due to TurNP + EP treatment indicates that MDA-MB231 cells are susceptible to the treatment because these enzymes are known to overexpress in various cancers^51–53^. These results were validated by demonstrating a good correlation between mRNA and protein level expressions of LDHB and ENO1 for the different treatments.

The upregulated glycolytic enzymes were either upstream or localized in the mitochondrion. Among these, the upregulated PDC complex enzymes (DLAT, DLD, PDHA1, and PDHB) indicate the restoration in PDC activity, which can revert the Warburg metabolic phenotype by redirecting pyruvate metabolism to the mitochondria and enhance apoptosis^54^. The upregulation of TCA cycle and OXPHOS pathway proteins also indicates a shift towards the mitochondrial metabolism upon TurNP + EP treatment, with a larger dependency on oxidative energy substrates for energy production in MDA-MB-231 cells. The upregulation in several other pathways, such as FA degradation, FA metabolism, amino acid (valine, leucine, isoleucine, and lysin) degradation, peroxisome, pyruvate metabolism, and aromatic amino acid (tryptophan) metabolism can produce pyruvate and TCA cycle intermediates, like acetyl COA and oxaloacetate to fuel the TCA cycle. The increased OXPHOS could also be a stress response as cells try to generate energy through alternate sources upon downregulation of glycolysis. Increased OXPHOS activity can also generate ROS to activate cell death pathways, as we showed previously for EP application with cisplatin and curcumin in MDA-MB-231 cells^20,55^. The peroxisome pathway, which involves breaking down the FA for the membrane regeneration can also generate H_2_O_2_ to trigger apoptosis^56^. We also measured the H_2_O_2_ levels, validating that TurNP + EP treatment causes the oxidative stress in these cells to cause cell death, correlating well with previous results^20,55^.

In summary, our results provide preliminary novel evidences and insights into the anticancer effects of ECT with TurNP against MDA-MB-231 cells. The combined TurNP + EP suppressed key proteins implicated in cancer cell proliferation, differentiation, migration, survival, and evasion of cell death and apoptosis. These proteins were involved in multiple pathways, such as PI3K-Akt signaling and glycolysis and their downregulation altered the metabolic profile of the MDA-MB-231 cells. These results suggest that the suppression of glycolytic metabolism in TNBC could be a potential therapeutic avenue against TNBC.

## Methods

### The Cells

Both MDA-MB-231 (TNBC) and MCF10A were grown and maintained at 37 °C, 70–80% humidity, and 5% CO_2,_ as previously^55^. Dulbecco’s Modified Eagle Medium (DMEM) (Gibco, USA) supplemented with 1% Penicillin-Streptomycin and 10% FBS (Corning, USA) was used for MDA-MB-231, while DMEM:F12 (1:1) (Gibco) supplemented with 5% horse serum (Atlanta Biologicals), 20 ng/ml human EGF (Sigma-Aldrich, USA), 0.5 mg/mL hydrocortisone (Sigma-Aldrich), 100 ng/mL cholera toxin (Sigma-Aldrich), 10 mg/mL bovine insulin (Sigma-Aldrich), 100 IU/mL penicillin and 100 mg/mL streptomycin was used for MCF10A. Using trypsin, cells were detached and were centrifuged at 1000 rpm for 5 min at 4 °C and were resuspended in fresh media at 1×10^6^ cells/mL for treatment.

### TurNP preparation

TurNP synthesis was carried out with some modification^57,58^. First, an aqueous extract from the dried Turmeric tuber (*Curcuma longa*) was prepared. The 2 g of the manually ground fine powder from Turmeric tuber was added to 20 mL of sterile distilled water (10% w/v), and was incubated overnight at 40 °C and 100 rpm (Scigenics biotech pvt. Ltd, India). The solution was boiled for 1 minute in the microwave and incubated for 10 minutes at 1000 rpm at 25 °C. The supernatant was filtered through Whatmann No:1 filter paper, and was concentrated to a final volume of 5 mL^59,60^.

For TurNP synthesis, 5mL of filtered Turmeric extract was added to 45 mL of 1 mM AgNO_3_ solution, and incubated for 3 days in dark at 100 RPM at 40 °C. 1 mM AgNo_3_ solution was used as control. The TurNP synthesis was observed by UV–vis spectroscopy between 300 nm to 650 nm. The TurNP synthesis was confirmed by a peak at 440 nm in UV–vis spectrum, and visually by the change in color from yellow to brown (not in AgNO_3_ control). After confirmation, the extract was centrifuged at 15,000 rpm for 20 min at 4 °C to obtain pellet. The pellet was washed once in sterile water and was dried to obtain TurNP powder used in the study.

### TurNP treatment

A stock solution of 1 mg/mL was prepared by suspending the TurNP into sterile double-distilled water with 10% DMSO, and sonicated for 2 minutes at 5% intensity for homogenization. The required volume from the stock solution was added into the cell suspension to achieve the desired final treatment concentration (5, 10, 15, and 25 µg/mL) of TurNP.

### The electrical pulse application

Eight, 600–1200 V/cm, 100 μs unipolar, square wave pulses at 1 Hz repetition rate were applied using a BTX-ECM830 electroporator (Genetronics Inc., USA). For this, 600 μL cell suspension (1 × 10^6^ cells/mL) with or without TurNP in BTX electroporation cuvettes (4 mm gap) was used, as previously^20,55^. No electrical pulses were applied to Ctrl and TurNP. After treatment, cells were transferred and cultured with fresh-media for 12 h for various assays.

### Viability assay

Following treatment, cells were transferred to 96-well plates (MDA-MB-231: 20,000 cells and MCF 10 A: 10,000 cells (as suggested by manufacturer’s protocol)) with fresh-media, as previously^20,55^. The cells were incubated for 12 h to assess the metabolic activity using RealTime-Glo MT Cell Viability Assay (Promega, USA), as per manufacturer’s protocol. Synergy LX Multi-Mode Reader (BioTek Instruments, USA) was used to record luminescence (Lum) at 1 s integration time. The sample Lum values were normalized with Ctrl to quantify viability using equation (1).

1 $$Cell\,Metabolic\,Activity\,( \% )=\frac{{\rm{Lum}}\,{\rm{value}}\,{\rm{of}}\,{\rm{sample}}}{{\rm{Lum}}\,{\rm{value}}\,{\rm{of}}\,{\rm{control}}}\times 100$$

### Hydrogen peroxide assessment

MDA-MB-231 cells were transferred to 96-well plates (20,000 cells/well) after treatment, with 60 μL fresh-media and were incubated for 12 h, as described previously^20,55^. At 7 h, 20 μL of H_2_O_2_ substrate solution from ROS-Glo H_2_O_2_ assay kit (Promega) was added to cells and incubated for 5 h more. At 12 h, cells were incubated for 20 min after addition of ROS-Glo™ detection solution and the Lum was measured at 0.5 s integration. Synergy LX Multi-Mode Reader was used for this purpose.

### Metabolites detection

MDA-MB-231 cells were incubated with fresh media in 6-well plates for 12 h, after treatment, as described previously^20^. After 12 h, the supernatant as well as cell were collected by scraping. Then, they were washed twice with ice-cold 1×PBS, resuspended in 1×PBS and counted using a Cellometer (Nexcelom Bioscience, USA). 35,000 cells were then used as per manufacturer’s protocol to detect the metabolites, such as glucose uptake (Glucose Uptake-Glo Assay, Promega), lactate (Lactate-Glo™ Assay, Promega), glutamine, and glutamate (Glutamine/Glutamate-Glo Assay, Promega).

### Real time quantitative PCR

MDA-MB-231 cells were incubated with fresh media in 6-well plates (600,000 cells/well) for 12 h, after treatment, as previously^55^. After 12 h, all cells were collected by trypsinization, washed thrice with ice-cold 1 × PBS. The RNeasy Mini Kit (QIAGEN, USA) was used to extract total RNA and it was quantified using Synergy LX Multi-Mode Reader. The cDNA was synthesized using iScript cDNA Synthesis Kit (Bio-Rad Laboratories, USA) and was quantified using Synergy LX Multi-Mode Reader. The equal amount of cDNA was then used with SYBR Green I Master (Roche, USA) and appropriate primers for qPCR reactions in CFX Connect Real-Time PCR system (Bio-Rad Laboratories) to measure mRNA levels. The parameters were as follow: 10 min at 95 °C, [15 sec at 95 °C, 10 sec at 60 °C, 30 sec at 72 °C] × 54 cycles, 10 sec at 95 °C, 60 sec at 65 °C, 1 sec at 97 °C. The relative mRNA levels were determined using the ∆∆Cq technique using 18 S ribosomal RNA (18 s rRNA) as the internal control. The primer sequences were: LDHB: upstream 5′-CCT CAG ATC GTC AAG TAC AGT CC-3′, and downstream 5′-ATC ACG CGG TGT TTG GGT AAT-3′. ENO1: upstream 5′-AAA GCT GGT GCC GTT GAG AA-3′, downstream 5′-GGT TGT GGT AAA CCT CTG CTC-3′. 18 s rRNA: upstream 5′-CCA GTA AGT GCG GGT CAT AAG-3′, and downstream 5′-GGC CTC ACT AAA CCA TCC AA-3′.

### Proteomics Studies

Following treatment, MDA-MB-231 cells were incubated in 6 well plates (600,000 cells/well) containing 2 mL of fresh-media and were cultured for proteomics experiments. The following methodology was adopted for sample preparation, mass spectroscopy run, and data analysis, as previously:^20,55^

#### Sample preparation for mass spectrometry analysis

After treatments, MDA-MB-231 cells were incubated in 6 well plates (600,000 cells/well) containing 2 mL of fresh-media. After 12 h, cells were collected by scraping, washed thrice in ice-cold 1×PBS and re-suspended in 4 M urea. Protein extraction and proteomics sample preparation was done, as described previously^20,55^. In brief, cells were homogenized in Precellys 24 Bead Mill Homogenizer (Bertin Corp., USA) and extracted proteins were precipitated overnight at −20 °C with pre-chilled (−20 °C) acetone. Precipitated proteins were dissolved in 8 M urea and protein concentration was estimated using BCA assay. Protein (50 µg) from each sample was digested overnight with Trypsin/Lys-C Mix (Promega) following reduction of disulfide bonds with dithiothreitol and cysteine alkylation with iodoacetamide. Peptides were desalted using C18 micro spin columns (The Nest Group Inc., USA) prior to LC-MS/MS analysis^20,55^.

#### LC-MS/MS data collection

LC-MS/MS data were collected on Q-Exactive (QE) High Field (HF) Hybrid Quadrupole Orbitrap MS (Thermo Fisher Scientific) coupled with an UltiMate 3000 RSLCnano HPLC and a Nano-spray Flex ion source (Thermo Fisher Scientific) using a standard data-dependent acquisition. Peptides (1 µg) were loaded onto a trap column (300 µm ID × 5 mm, 5 µm 100 Å PepMap C18 medium) and then separated on a 15-cm long Acclaim™ PepMap™ (75 µm, 3μm 100 Å PepMap C18 medium, Thermo Fisher Scientific) analytical column. All the MS measurements were performed in the positive ion mode using 120 min LC gradient method as described elsewhere^61,62^, MS data were collected using Top20 data dependent MS/MS scan method.

#### Data analysis

LC-MS/MS data were analyzed using MaxQuant software (version.1.6.1.0)^63,64^ against the Uniprot Homo sapiens protein database (http://www.uniprot.org; updated on March, 2019), as previously^20,55,65^. All default settings were used except the following settings: precursor mass tolerance was 10 ppm, enzyme specificity was Trypsin/P and Lys-C with up to 2 missed cleavages, variable modification was oxidation of methionine (M); fixed modification was carbamidomethylation of cysteine (C); false discovery rate (FDR) of peptides and proteins was 0.01. Unique plus razor peptides were used for protein quantitation. Proteins were quantified using LFQ intensity. Post search data analysis was performed as described previously^20,55^. Zero LFQ values were imputed with 983600, half of the lowest LFQ value (1967200) observed across three treatments. The protein fold-change was calculated by subtracting the average log2 values [Δlog2 (LFQ intensity)] between proteins from each comparison group. Proteins with fold-change of | Δlog2 | >0.5, and P < 0.05 (Student’s unpaired, two-tailed, t test) were considered significant.

#### Enrichment and string interaction analysis

Significant proteins were compared among treatments using KEGG database^66^ in DAVID 6.8^67,68^. The proteins from KEGG pathway analysis were uploaded to the Cytoscape 3.6.1 software^69^ and matched using the WikiPathway app (beta), with the degree of shading representing the fold change. GO enrichment analysis was performed using Genecodis^70^, using total 2426 proteins as background. STRING^71^ was used to visualize the interaction and functional enrichment with minimum required interaction score as highest confidence (0.9). An MCL clustering analysis with 3 as inflation parameter was run on the network nodes to cluster them in different groups.

### Statistical analysis

One-way ANOVA was used to calculate statistical significance for cell viability assays, coupled with Tukey’s multiple comparison test, as previously^55^. Prior to ANOVA analysis, the data were checked against normality and homoscedasticity assumptions. Tukey’s test tags each treatment with a letter or a group of letters to indicate their significance. The same letter or the same groups of letters indicate that they are not significantly different. The different letters or different group of letters indicate that they are significantly different (P < 0.05).

Statistical significance for proteomics data (log2 transformed), and all other experimental data was calculated using Student’s unpaired, two-tailed, t-test.

All experiments were performed in triplicates or more.

## Supplementary information

Supplementary information.

Supplementary tables.

## Data Availability

RAW proteomics data files, parameters used, and LC–MS/MS methodology and statistics, and the other datasets generated and/or analyzed during the current study are available from the corresponding author on reasonable request.
