# Supplementary material for: High-throughput, Label-Free Quantitative Proteomic Studies of the Anticancer Effects of Electrical Pulses with Turmeric Silver Nanoparticles: an in vitro Model Study
Source: Sci Rep. 2020 Apr 29;10:7258. doi: 10.1038/s41598-020-64128-8 (PMC7190727; doi:10.1038/s41598-020-64128-8)
Supplement: Supplementary file 1 — Supplementary information. [file 41598_2020_64128_MOESM1_ESM.docx]

**High-throughput, Label-Free Quantitative Proteomic Studies of the Anticancer Effects of Electrical Pulses with Turmeric Silver Nanoparticles: an *in vitro* Model Study**

Lakshya Mittal^1,^^†^ , Ignacio G. Camarillo^2,3,†^, Gowri Sree Varadarajan^4^, Hemalatha S^5^, Uma K. Aryal^6,7^, and Raji Sundararajan^1,*^

^1^School of Engineering Technology, Purdue University, West Lafayette, IN, 47907, USA

^2^Department of Biological Sciences, Purdue University, West Lafayette, IN, 47907, USA

^3^Purdue Center for Cancer Research, Purdue University, West Lafayette, IN, 47907, USA

^4^Division of High Voltage Engineering, Dept. of Electrical & Electronics Engineering, College of Engineering, Anna University, Guindy, Chennai, TN, 600025, India

^5^School of Life Sciences, B. S. Abdur Rahman Crescent Institute of Science & Technology, Chennai, TN, 600048, India

^6^Purdue Proteomics Facility, Bindley Bioscience Center, Purdue University, West Lafayette, IN, 47907, USA

^7^Dept. of Comparative Pathobiology, College of Veterinary Medicine, Purdue University, West Lafayette, IN, 47907, USA

**^†^**Co-First Authors

*****Corresponding author **(**[raji@purdue.edu](mailto:raji@purdue.edu))

***Supplementary Tables***

Table S1: Peptide Sequence and Raw Intensity.

Table S2: Proteins Identified in Biological Replicates are Listed with Their Corresponding Intensities and MS/MS (Spectral) Counts.

Table S3: Proteins which are identified to be significantly (p<0.05) regulated (|Fold change|>0.5) in various pairwise comparisons: TurNP vs Ctrl, TurNP+EP vs TurNP, TurNP+EP vs Ctrl, TurNP+EP vs EP, EP vs Ctrl, and EP vs TurNP. The Intensity is a log2 transformed LFQ intensity.

Table S4: GO enrichment for significantly regulated proteins in various comparisons using Genecodis: TurNP vs Ctrl, TurNP+EP vs TurNP, TurNP+EP vs Ctrl, TurNP+EP vs EP, EP vs Ctrl, and EP vs TurNP.

Table S5: Enriched pathways for significantly regulated proteins in various comparisons using DAVID 6.8: TurNP vs Ctrl, TurNP+EP vs TurNP, TurNP+EP vs Ctrl, TurNP+EP vs EP, EP vs Ctrl, and EP vs TurNP.
